# Masticatory muscle index for indicating skeletal muscle mass in patients with head and neck cancer

**DOI:** 10.1371/journal.pone.0251455

**Published:** 2021-05-10

**Authors:** Sheng-Wei Chang, Yuan-Hsiung Tsai, Cheng-Ming Hsu, Ethan I. Huang, Geng-He Chang, Ming-Shao Tsai, Yao-Te Tsai

**Affiliations:** 1 Department of Radiology, Chang Gung Memorial Hospital, Chiayi, Taiwan; 2 Department of Otorhinolaryngology-Head and Neck Surgery, Chang Gung Memorial Hospital, Chiayi, Taiwan; IRCCS Ospedale Policlinico San Martino, ITALY

## Abstract

**Background:**

A typical assessment for sarcopenia involves the use of abdominal computed tomography (CT) for calculating the skeletal muscle index (SMI) at the level of the third lumbar vertebra (L3). However, abdominal CT is not regularly performed on patients with head and neck cancer (HNC). We investigated whether masticatory SMI (M-SMI) measurements based on head and neck CT scans can be used to conduct sarcopenia assessments by evaluating whether M-SMI is correlated with L3-SMI.

**Methods:**

Abdominal and head and neck CT images of patients with trauma (n = 50) and HNC (n = 52) were analyzed retrospectively. Both manual delineation and threshold selection methods were used to measure cross-sectional areas of masticatory muscles and those of muscles at the L3 level on CT images. Muscle cross-sectional areas were normalized to height squared to calculate SMI, and a multivariate linear regression model was established to evaluate the correlation between the M-SMI and L3-SMI. Receiver operating characteristic curve analysis was used to assess the ability of the M-SMI to identify sarcopenia, and Cox logistic regression was used to identify predictors of sarcopenia.

**Results:**

Patients with HNC had significantly lower M-SMI and L3-SMI than did patients with trauma (*p* = 0.011 and 0.03, respectively). M-SMI and L3-SMI were strongly correlated (*r* = 0.901, *p* < 0.001); in the multivariate model that included sex, the correlation was stronger (*r* = 0.913, *p* < 0.001). The associations of sarcopenia with a lower M-SMI (*p* < 0.001), male sex (*p* = 0.028), and advanced age (*p* = 0.011) were significant, and multivariate logistic analysis demonstrated that an M-SMI of <5.5 was an independent predictor of sarcopenia (hazard ratio = 5.37, *p* < 0.001).

**Conclusions:**

M-SMI assessment in routine head and neck CT scans is feasible and can be an alternative for detecting sarcopenia in patients with HNC.

## Introduction

Cachexia is common in patients with cancer [1], and associated muscle wasting and severe malnutrition are reportedly higher in patients with head and neck cancer (HNC) than in those with other cancers [2]. Progressive depletion of skeletal muscle mass (SMM), namely sarcopenia, can lead to adverse clinical outcomes in patients with cancer; these adverse outcomes include increased incidences of requiring inpatient rehabilitation care and developing postoperative complications, prolonged hospitalization, and chemotherapy-induced toxicity; as a consequence, patients tend to have poor cancer prognosis [3–7]. Studies have revealed that in patients with cancer, an increased neutrophil-to-lymphocyte ratio (NLR), male sex, a decreased lymphocyte count, and an Eastern Cooperative Oncology Group score of >2 are significant predictors of sarcopenia [8–10]. Moreover, the increased NLR in patients with sarcopenia indicates that inflammation may play a role in the development of sarcopenia [11]. In oncological research, sarcopenia has frequently been evaluated using the skeletal muscle index (SMI) at the level of the third lumbar vertebra (L3-SMI) as a quantitative indicator of whole-body SMM, calculated as the skeletal muscle cross-sectional area (CSA) normalized by height squared and obtained using computed tomography (CT) or positron emission tomography CT (PET-CT) images [12]. The use of sex-specific L3-SMI cutoffs has also been reported to categorize patients as having sarcopenia [13, 14]. Nevertheless, despite the increased risk of sarcopenia in patients with HNC, they do not necessarily undergo routine whole-body CT or PET-CT, particularly in early-stage disease, rendering the L3-SMI not always applicable. Given these limitations, studies have proposed conducting muscle CSA assessment at the level of the third cervical vertebra (C3); such an assessment can be based on pretreatment head and neck CT images, offering a cost-effective alternative to L3 muscle measurement in patients with HNC [12, 15] and a feasible imaging marker to predict prognosis in patients with oral cavity cancer [16]. However, frequent metastatic cervical lymphadenopathy, especially with extranodal extension, or previous neck dissection with muscle sacrifice may hinder the delineation of the CSA of cervical muscles in patients with locally advanced or recurrent HNC [12].

The CSA of masticatory muscle reflects the swallowing function and nutritional status of patients with cancer [17], and a study demonstrated that masticatory muscles are significantly associated with systemic nutritional biomarkers and may have more nutritional importance than do the paraspinal muscles [18]. Masseter muscles CSA measurements obtained using head and neck CT are also validated indicators of sarcopenia in patients with trauma [19, 20]. However, this marker has not been examined in relation to patients with HNC according to our literature review. Our study investigated whether masticatory muscle CSA can be assessed using conventional head and neck CT, and we compared the masticatory SMI (M-SMI) assessed at the mandibular notch level with the L3-SMI to determine whether the M-SMI can be an alternative indicator of SMM in patients with HNC. The present study enrolled 52 patients with newly diagnosed HNC who underwent pretreatment whole-body PET-CT and 50 patients in the trauma department without a cancer history (considered healthy controls) who received whole-body CT screening. If the M-SMI obtained from head and neck CT is a reliable whole-body SMM marker, then this value may facilitate routine assessment for sarcopenia in patients with HNC for oncology research and clinical practice.

## Materials and methods

### Ethical considerations

Our current study complied with the principles of the Declaration of Helsinki and received approval from Chang Gung Memorial Hospital’s Institutional Review Board (number: 201901763B0). All CT image files and clinical records were anonymized; therefore, the requirement to obtain informed patient consent was waived.

### Study design and patient populations

In this single-center retrospective cohort study, whole-body CT scans conducted between January 1, 2014, and December 31, 2018, at Chiayi Chang Gung Memorial Hospital were randomly selected from two retrospectively assigned groups of patients. The first group comprised 57 randomly selected patients with HNC undergoing whole-body PET-CT scans as a part of radiotherapy planning. The other group consisted of 53 patients with trauma who underwent whole-body CT at the Department of Emergency Medicine and were considered to be otherwise healthy controls. Patients were eligible for enrollment if they (1) were aged >18 years, (2) were treated for HNC or trauma at our hospital, and (3) had complete pretreatment data and imaging studies. Patients with any of the following conditions were excluded: (1) a history of cancer, concurrent malignancy, or distant metastasis at presentation (n = 3) or (2) missing or incomplete preoperative CT images or images with impaired quality (n = 5). The electronic medical records of all patients were retrospectively reviewed and documented. The clinical stage of cancer according to the American Joint Committee on Cancer’s Cancer Staging Manual (Eighth Edition) was recorded, and the Charlson Comorbidity Index (CCI) was used to categorize the underlying conditions [21].

### CT image processing and interpretation

All whole-body CT images were obtained before treatment as Digital Imaging and Communications in Medicine (DICOM) 3.0 files from the Chiayi Chang Gung Memorial Hospital image archiving and communication system. CT was conducted craniocaudally on a multidetector CT scanner (Biograph TruePoint, Siemens, Knoxville, TN, USA), and whole-body CT images were acquired without contrast medium by using the following parameters: 120-kV tube voltage, 50-mA tube current, 0.5-s tube rotation time, 0.8 pitch, and 5-mm slice thickness for attenuation correction and image fusion. Axial images were used for image selection, and the standard procedure of scrolling in a caudal–cephalad direction through the mandibular ramus and L3 vertebra was followed. The reference point for masticatory muscle CSA measurement in the axial CT images was the mandibular notch, which was selected because it is easy to identify on CT images and is a crucial surgical landmark with prognostic significance in HNC [22, 23]. We selected the first CT slides displaying the bilateral mandibular notches to assess bilateral masticatory muscle CSA (cm^2^) and the entire L3 vertebral arch and transverse processes to assess L3 muscle measurements. All CSAs of the bilateral masticatory muscles, including the pterygoid and masseter muscles, can be measured using the same slice without interference from lymphadenopathy or primary tumor extension. Among the masticatory muscles, the temporalis muscle at the mandibular notch level is mostly temporalis tendon rather than skeletal muscle; therefore, the CSA of the temporalis muscle was not taken into account in this measurement protocol.

**Fig 1:**
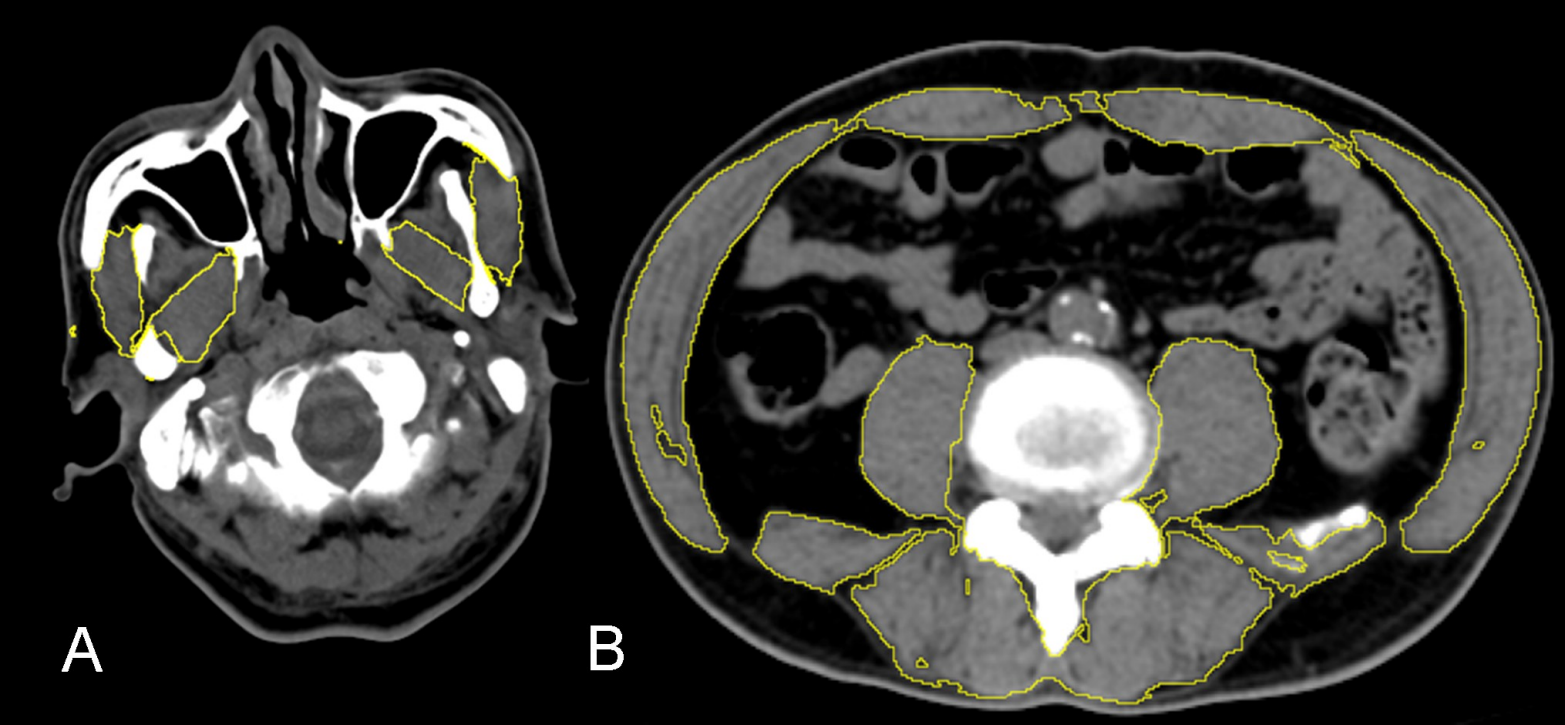
ImageJ delineation of L3 and masticatory muscles with the following threshold setting: −29 to +150 Hounsfield units. Both images have a clinical soft tissue window setting with a 350-HU width and a 40-HU level. (A) Mandibular notch–level masseter and pterygoid muscle delineations. (B) L3-level muscle CSA delineations.

Analysis of masticatory and L3 muscle CSAs as well as image selection were performed as reported previously [12, 13, 15] by using the freeware package ImageJ (version 1.52a; Wayne Rasband, National Institutes of Health, USA). As presented in Fig 1, threshold selection was used to obtain the CSA of pixels with radiodensity ranging from −29 to 150 Hounsfield units (HUs) for skeletal muscle identification [12]. After two independent radiologists (SWC and WST) performed manual delineation, the sum of the demarcated pixels was obtained automatically as the CSA of the manually selected region. Manual delineation and threshold selection were performed to compare the target SMIs obtained using each approach, and all the CSA values from both methods were then normalized by height squared to calculate the SMI (cm^2^/m^2^) for the subsequent analysis. Interobserver reliability was assessed using the intraclass correlation (ICC) in a two-way mixed model. In addition, we defined low SMM and sarcopenia on the basis of the sex-specific L3-SMI cutoffs (≤38.9 and ≤52.4 cm^2^/m^2^ for women and men, respectively) reported by Prado et al. [14]. The primary outcome was the correlation between the M-SMI and L3-SMI, and the secondary outcome was the discrimination ability of the M-SMI for sarcopenia.

### Statistical analysis

Percentages were used to express categorical variables; by contrast, to indicate continuous variables, we used means with standard deviations and medians with interquartile ranges for data distributed normally and those not distributed normally, respectively. The Kolmogorov–Smirnov test was used to test normality; age, weight, M-SMI, and L3-SMI were normally distributed, whereas body mass index (BMI) was not. For comparing two patient groups’ characteristics, the Mann–Whitney *U* test, independent *t* tests, and Pearson’s *χ*^2^ tests were used for skewed, normally distributed, and categorical variables, respectively. The correlation between L3 and masticatory muscle measurements was modeled using Pearson correlation coefficients (*r*) and linear regression analysis to determine the association between the M-SMI and L3-SMI. The agreement between the predicted and measured L3-SMI was examined by generating Bland–Altman plots [24]. Additionally, receiver operating characteristic curve analysis was performed to assess the diagnostic performance and optimal cutoff value of the M-SMI for the diagnosis of sarcopenia relative to the reported cutoff values of the L3-SMI (≤38.9 cm^2^/m^2^ for women and ≤52.4 cm^2^/m^2^ for men) [14]. A Cox logistic regression analysis was used to identify the potential predictors of sarcopenia, and the parameters applied in the univariate logistic analysis were the M-SMI, age, body mass index (BMI), NLR, and CCI. We used SAS version 6.3 (SAS Inc., Cary, NC, USA) for all analyses, and *p* < 0.05 indicated statistical significance.

## Results

Baseline characteristics of the 102 patients, of whom 50 had trauma and 52 had HNC (age, 67.2 ± 16.4 years), are listed in Table 1. The groups had similar body weight and BMI distributions, but the patients with HNC were primarily male (*p* = 0.028) and significantly older than the patients with trauma (*p* = 0.012). In addition, patients with HNC had significantly lower M-SMI and L3-SMI (*p* = 0.011 and 0.03, respectively). Of the patients with HNC, 11 (21.2%) had cervical lymph node metastasis and more than half (n = 29, 55.8%) had stage III or IV disease. Tumor locations included the oral cavity (32 cases, 61.5%), hypopharynx (11 cases, 21.2%), oropharynx (6 cases, 11.5%), and larynx (3 cases, 5.8%).

**Table 1:** Baseline demographics of the patients with trauma and head and neck cancer.

Variable	Trauma	HNC	*p*-value
	(n = 50)	(n = 52)	
Sex			0.028 ^a^
Female	11 (22.0)	4 (7.7)	
Male	39 (78.0)	48 (92.3)	
Age (year)	59.6 (9.9)	72.4 (7.1)	0.012^b^
Weight (kg)	68.1 (15.6)	65.7 (10.2)	0.277^b^
BMI (kg/m^2^)	25.7 (22.5−28.7)	24.2 (21.6−26.8)	0.314^c^
SMI (cm^2^/m^2^)			
M-SMI	5.6 (2.1)	4.7 (1.4)	0.011^b^
L3-SMI	47.1 (16.2)	42.1 (11.9)	0.03^b^
Overall stage	n.a.		n.a.
Early (I + II)		23 (44.2)	
Advanced (III + IV)		29 (55.8)	
T classification	n.a.		n.a.
T 1−2		30 (57.6)	
T 3−4		22 (42.4)	
N classification	n.a.		n.a.
N0		41 (78.8)	
N1−3		11 (21.2)	
Tumor location	n.a.		n.a.
Oral cavity		32 (61.5)	
Hypopharynx		11 (21.2)	
Oropharynx		6 (11.5)	
Larynx		3 (5.8)	
CCI			0.106 ^a^
0	43 (86)	38 (73.1)	
≥ 1	7 (14)	14 (26.9)	

Abbreviation: BMI, body mass index; CCI, Charlson Comorbidity Index; HNC, head and neck cancer; L3-SMI, skeletal muscle index at third lumbar level; M-SMI, masticatory skeletal muscle index; n.a., not applicable; SMI, skeletal muscle index.

The age, weight, and SMI are shown as the mean (standard deviation), BMI was shown as median (interquartile range), and other variables are shown as n (%).

a. Mann-Whitney U-test.

b. Independent t-test.

c. Pearson’s *χ*^2^ tests.

Table 2 presents the results of image analysis. Measurements of target skeletal muscles were accessible in all patients (S1 Appendix). High interobserver reliability between the two radiologists was observed for both SMIs (ICC = 0.99 for the L3-SMI and ICC = 0.997 for the M-SMI, both *p* < 0.001). The M-SMIs were consistent between the manual delineation and threshold selection approaches (0.95%, *p* = 0.304, Table 2), whereas the manually delineated L3-SMIs were significantly overestimated compared with the threshold-selected L3-SMIs (6.05%, *p* = 0.011, Table 2). Because manual delineation resulted in these overestimated results, which may have been caused by muscle fattening, we used the M-SMI and L3-SMI obtained from threshold selection in subsequent analysis. Additional analyses revealed that differences in both the M-SMI and L3-SMI were nonsignificant between patients with early (stage I/II) and advanced (stage III/IV) stages.

**Table 2:** Comparison between skeletal muscle index determined by manual delineation and threshold selection on L3 and masticatory muscle.

Variable	Manual Delineation		Threshold Selection		*Diff* ^*a*^ *(%)*	*p* value^b^
	Mean	SD	Mean	SD		
M-SMI	5.2	1.6	5.1	1.7	0.95%	0.304
Masseter-SMI	2.2	0.7	2.1	0.6	1.00%	0.258
Pterygoid-SMI	3.1	0.8	2.9	0.7	0.92%	0.341
L3-SMI	47.4	10.9	44.7	9.3	6.05%	0.011

Unit: cm^2^/m^2^.

Abbreviation: Diff, difference; SD, standard deviation; SMI, skeletal muscle index; L3-SMI, skeletal muscle index at third lumbar level; M-SMI, masticatory skeletal muscle index.

^a^ Diff was defined as skeletal muscle index of (Manual delineation–Threshold Selection)/Threshold selection ×100%.

^*b*^ *p* value is based on Independent Student t-test.

We could analyze the trauma and HNC groups as a single entity because no significant intergroup interaction was observed among these patients. Table 3 presents the regression analysis results. The Pearson correlation analysis indicated that the M-SMI and L3-SMI were correlated significantly (*r* = 0.901, *p* < 0.001, Table 3). Because of this strong correlation, the following prediction formula was established:

$$L3‐SMI\;(cm^{2}/m^{2})=7.21\times M‐SMI\;(cm^{2}/m^{2})+7.56$$

**Table 3:** Univariate and multivariate regression analysis.

Method	Covariates	r	*p* value*
Univariate model	M-SMI	0.901	<0.001
Prediction rule	L3-SMI = 7.21x M-SMI +7.56		
Multivariate model		0.913	<0.001
	M-SMI (continuous)	r_partial_ = 0.898	<0.001
	Sex (male = 1)	r_partial_ = −0.179	0.037
	Presence of HNC (cancer = 1)	r_partial_ = −0.094	0.357
	Age (continuous)	r_partial_ = −0.051	0.617
	BMI (continuous)	r_partial_ = 0.016	0.872
Prediction rule	L3-SMI = 7.34 × M-SMI– 3.28 × Sex (male = 1, female = 0) + 9.01		

Abbreviation: BMI, body mass index; L3-SMI, skeletal muscle index at third lumbar level; M-SMI, masticatory skeletal muscle index.

We introduced several clinical variables to the regression model and used the entry method to analyze the correlation between the L3-SMI and the enrolled variables, including the M-SMI, sex, the presence of HNC, age, and BMI. The pooled Pearson correlation coefficient of the multivariate model was 0.913 (*p* < 0.001), and the established model can be expressed as

$$L3‐SMI\;(cm^{2}/m^{2})=7.34\times M‐SMI\;(cm^{2}/m^{2})–3.28\times Sex\;\left(male=1,\;female=0\right)+9.01$$

Although sex independently predicted the L3-SMI (*r* = −0.179, *p* = 0.037), the M-SMI remained the strongest predicting factor in the multivariate analysis (*r* = 0.898, *p* < 0.001). Correlations of age, BMI, and HNC status with the L3-SMI were nonsignificant.

**Fig 2:**
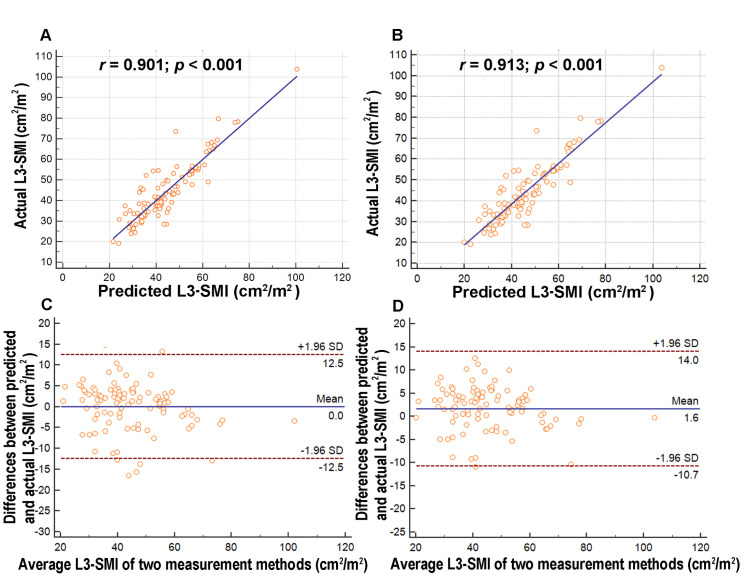
Analysis of L3-SMI estimates obtained using the M-SMI only and using the multivariate model. A significant correlation was observed between the L3-SMI measurements and predictions in (A) the univariate model and (B) the multivariate model. Bland–Altman plots exhibited reasonable agreement in the (C) univariate and (D) multivariate models. L3 = third lumbar vertebra; M-SMI = masticatory skeletal muscle index; SD = standard deviation; SMI = skeletal muscle index.

Regression analysis revealed a significant correlation between the L3-SMI measurements and predictions in the univariate model (Fig 2A) and multivariate model (Fig 2B). We also performed Bland–Altman analysis to compare the L3-SMI measurements and predictions. The mean difference between the L3-SMI measurements and predictions in the univariate (Fig 2C) and multivariate (Fig 2D) model were 0 and 1.6 (cm^2^/m^2^). The interval between two standard deviations was 25(cm^2^/m^2^) and 24.7(cm^2^/m^2^) in the univariate and multivariate models, respectively, suggesting reasonable agreement between the L3-SMI measurements and predictions.

Table 4 presents a comparison of the clinicopathological features between patients with and without sarcopenia according to the sex-specific L3-SMI cutoffs [14]. The sarcopenia and nonsarcopenia groups consisted of 72 (70.6%) and 30 (29.4%) patients, respectively, and associations were observed for sarcopenia with male sex (*p* = 0.028), older age (*p* = 0.011), low L3-SMI (*p* < 0.001), and low M-SMI (*p* < 0.001), including low SMI for both the masseter (*p* < 0.001) and pterygoid (*p* = 0.001) muscles.

**Table 4:** Clinicopathological characteristics in patients with and without sarcopenia (n = 102).

Variable	Sarcopenia	Non-sarcopenia	*p* value
	(n = 72)	(n = 30)	
Sex, n (%)			0.028^a^
Male	65 (90.3%)	22 (73.3%)	
Female	7 (9.7%)	8 (26.7%)	
Age (year), mean (SD)	69.7 (15.5)	61.0 (17.2)	0.014^b^
BMI (kg/m^2^), median (IQR)	24.6 (21.3−26.6)	27.1 (20.5−29.5)	0.073^a^
NLR, median (IQR)	2.56 (2.30−3.15)	2.45 (2.10−2.77)	0.197^a^
CCI			0.223^a^
0	49 (68.1%)	24 (80.0%)	
≥ 1	23 (31.9%)	6 (20.0%)	
L3-SMI (cm^2^/m^2^), mean (SD)	37.3 (7.7)	61.7 (11.7)	<0.001^b^
M-SMI (cm^2^/m^2^), mean (SD)	4.3 (1.1)	7.1 (1.6)	<0.001^b^
Masseter-SMI (cm^2^/m^2^), mean (SD)	1.9 (0.6)	3.2 (0.7)	<0.001^b^
Pterygoid-SMI (cm^2^/m^2^), mean (SD)	2.4 (0.8)	3.8 (0.9)	0.001^b^

Abbreviations: BMI, Body mass index; CCI, Charlson Comorbidity Index; IQR, interquartile range; L3-SMI, skeletal muscle index at third lumbar level; M-SMI, masticatory skeletal muscle index; SD, standard deviation.

a. Mann-Whitney *U*-test

b. Independent t-test.

By analyzing the receiver operating characteristic curves for sarcopenia, we determined the optimal M-SMI cutoff value to be 5.5 (sensitivity, 88.7%; specificity, 90.3%; area under the curve, 0.944; *p* < 0.001). Table 5 presents the outcomes of the multivariate and univariate analyses of potential predictors of sarcopenia. A univariate analysis revealed that an M-SMI of <5.5 (*p* < 0.001), and age of ≥75 years (*p* = 0.02) were found to be significantly associated with sarcopenia. In a multivariate analysis, an M-SMI of <5.5 (hazard ratio = 5.37, 95% CI: 1.28–8.51, *p* < 0.001) and age ≥75 years (hazard ratio = 6.54, 95% CI: 1.60–8.47, *p* = 0.005) were independent predictors of sarcopenia.

**Table 5:** Cox logistic regression analysis of independent predictors of sarcopenia.

Variable	Number of patients	Univariate analysis		Multivariate analysis	
		Hazard ratio (95% CI)	*p* value	Hazard ratio (95% CI)	*p* value
M-SMI					
≥ 5.5	25	Reference		Reference	
< 5.5	77	7.08 (1.41–10.31)	<0.001	5.37(1.28–8.51)	<0.001
BMI					
≥ 25	52	Reference			
< 25	50	1.39 (0.59–3.26)	0.438		
Age (years)					
<75	67	Reference		Reference	
≥75	35	3.57 (1.23–10.40)	0.020	6.54 (1.60–8.47)	0.005
NLR					
< 3	78	Reference			
≥ 3	24	1.979 (0.829–4.725)	0.125		
CCI					
0	73	Reference			
≥ 1	29	1.135 (0.842–1.331)	0.145		

Abbreviations: BMI = body mass index, CI = confidence interval, CCI = Charlson comorbidity index, M-SMI = masticatory skeletal muscle index, NLR = neutrophil-to-lymphocyte ratio.

## Discussion

Patients with HNC have high risks of malnutrition, dysphagia, and subsequently sarcopenia [2]. L3-SMI assessment through abdominal CT is the widely regarded standard for whole-body SMM and sarcopenia assessment [25]. However, performing abdominal CT for this purpose alone would be cost intensive for patients with HNC and expose them to excessive radiation. Studies have indicated that the quality of masticatory function is not only critical for mastication but also associated with handgrip strength, walking speed, and physical fitness parameters in elderly individuals [26, 27]. In patients with traumatic injury, a masseter muscle CSA 2 cm below the zygomatic arch can be used to predict mortality outcomes [18, 20]. Hwang et al. retrospectively reviewed 314 patients in an emergency department and demonstrated a significant association between masticatory muscle mass and systemic nutritional biomarkers [18]. These study results suggest that masticatory muscle mass is a potential indicator of nutritional status, physical activity level, and trauma-related prognosis. Moreover, we determined the feasibility of measuring the M-SMI with pretreatment head and neck CT images and evaluated the correlation between these M-SMI measurements and the L3-SMI in patients with HNC. According to our literature review, we are the first to analyze the association between L3 muscle and masticatory muscle measurements in patients with cancer. The results of this study reveal that the M-SMI is significantly correlated with the L3-SMI, suggesting that measuring the M-SMI of patients with HNC by using their head and neck CT images is a feasible alternative to whole-body SMM assessment. In addition, the patients with HNC had significantly lower M-SMI and L3-SMI values than those with trauma did, indicating that low SMM, which may be explained by tumor-related swallowing dysfunction and malnutrition, is a prevalent and noteworthy issue in this population [28]. We also observed significant associations of sarcopenia with male sex, old age, and low M-SMI, including the SMI of both the masseter and pterygoid muscles, in accord with the findings of previous studies [29, 30]. Multivariate analyses further revealed that an M-SMI of <5.5 and age of ≥75 years are independent predictors of sarcopenia. Our study also has therapeutic implications. Regular dietary counseling and nutritional intervention might help patients with HNC who are at nutritional risk or have low M-SMI to minimize muscle atrophy, maintain their body weight, and prevent severe malnutrition, thereby improving their survival outcomes and quality of life [31–33]. Overall, our results highlight the significant association between M-SMI and L3-SMI and indicate the informative role of M-SMI in detecting sarcopenia in patients with HNC.

A significant association between sarcopenia and male sex was observed in the present study, and several studies have demonstrated that the prevalence of sarcopenia differs between the sexes. Du et al. conducted a cross-sectional study of 631 patients and indicated that male patients were more likely to have sarcopenia and sarcopenic obesity than were female patients [30]. Wu et al. also reported that male sex was independently associated with sarcopenia in community-dwelling elderly adults in Taiwan [34]. Possible mechanisms underlying this sex-based difference in the prevalence of sarcopenia include 1) SMM loss tends to occur gradually with age in men, whereas this decline in SMM with age is less apparent among women [35] and 2) insulin-like growth factor-1 and testosterone levels decrease significantly with age among men, leading to the rapid loss of strength and SMM and an increased risk of sarcopenia [36]. In addition, we observed a significant association between older age and sarcopenia, which may be explained by a multifactorial process involving aging and oxidative stress, changes in dietary intake, a decline in physical activity, and hormonal changes with age [37]. Emerging evidence also suggests that the disruption of positive regulators of muscle hypertrophy (serum response factor and myocardin-related transcription factor A) may result in the development of skeletal muscle atrophy with age [38]. Systemic inflammation has been reported to be associated with loss of SMM and strength [39]. Moreover, a higher NLR, which is a systemic inflammation–based marker, has been reported to be an independent predictor of sarcopenia in patients with gastric and colorectal cancer [10, 40]. However, the correlation between NLR and sarcopenia was nonsignificant in our data. Degens proposed that the negative effects of systemic inflammation on muscle mass and strength may only become evident when they persist for a prolonged period and exceed a certain threshold [41], which may partially explain our results. The body of knowledge on this topic would benefit from studies using larger sample sizes to investigate the relationship between systemic inflammatory response and sarcopenia among patients with HNC.

Several studies have discussed the high sarcopenia prevalence and clinical impact in patients with HNC. Wendrich et al. demonstrated that in patients receiving chemoradiation for locally advanced HNC, sarcopenia increased treatment-related toxicity risk and severity [6]. Regarding survival outcomes, studies have indicated that cancer-specific survival and overall survival are reduced in patients with HNC whose CT images at presentation or after radiotherapy indicate low SMM [1, 42]. In an investigation of 85 patients who underwent surgery or radiotherapy for HNC, Nishikawa et al. proposed that surgery benefits patients with sarcopenia more than radiotherapy does [2]. In addition, better cancer-related outcomes were observed in patients with HNC who received definitive radiotherapy and adhered to regular dietetic counseling [43]. Although patients with HNC are known to develop sarcopenia and malnutrition, no consensus has been reached on the optimal method of evaluating the sarcopenic state in patients with HNC because abdomen CT, PET-CT, and dual-energy X-ray absorptiometry are not routinely performed for initial HNC diagnosis or in patient follow-up. Few studies have investigated whether head and neck muscle measurement in CT images is feasible for predicting whole-body SMM. Swartz et al. revealed a significant association between muscle CSAs at the level of C3 measured using images of head and neck CT and measurements of L3 muscles from images of abdominal CT [12]. Ufuk et al. retrospectively reviewed 159 patients with HNC and suggested that the SMIs of C2, C3, and C4 muscles and the sternocleidomastoid muscle (SCM) are adequate alternatives to the L3-SMI, and the SMI of the SCM is the most useful alternative to the L3-SMI in the detection of sarcopenia, regardless of gender differences [29]. A potential limitation of their technique is that cervical muscle assessment is frequently hindered by metastatic cervical lymphadenopathy or locoregional tumor extension, which have been reported to be identified in approximately 57% of patients at the time of HNC diagnosis [44]. SCM assessments are of particular concern because metastatic lymph nodes, especially in the presence of extranodal extension, frequently adhere to or invade the SCM, precluding the possibility of precise delineation of the SCM [45]. In the present study, the masseter and pterygoid muscles could be clearly defined in a single CT slice at the level of the mandibular notch; moreover, neither primary tumor invasion nor metastatic lymphadenopathy was observed at the bony notch level, making M-SMI assessment feasible in all patients. In patients with recurrent HNC, the assessment of the cervical paraspinal or SCM may be greatly influenced by prior neck dissection or radical tumor resection. We hypothesized that masticatory muscle assessment at the mandibular notch level would involve less interference from the aforementioned factors in patients with recurrent HNC; this hypothesis warrants further investigation in the future. Overall, we expanded the current knowledge on masticatory muscle measurement at the mandibular notch level in patients with HNC to detect low SMM and sarcopenia.

The correlation between cancer stage and SMM warrants attention because a large tumor burden may lead to higher metabolism and muscle depletion [46]. However, the relevant studies have yielded inconsistent results. Ufuk et al. indicated that the correlation between tumor stage and measured skeletal muscle CSA and indexes was weak and negative, respectively [29]. However, when the authors used sex-specific L3-SMI cutoffs (≤52.4 and ≤38.9 cm^2^/m^2^ for male and female patients, respectively) for diagnosing sarcopenia from images, tumor stage and sarcopenia were significantly correlated (*r* = 0.386, *p* < 0.001). By contrast, Swartz et al. observed no significant muscle depletion in advanced T or N stage cancer patients [12]. The current results revealed no significant differences in the M-SMI or L3-SMI between patients with early- and advanced-stage cancer. The relatively small sample size may have caused this lack of difference; therefore, large prospective studies are warranted to confirm these results.

By comparing manual delineation and threshold selection of the SMI with a radiodensity window of −29 to 150 HU, we revealed the overestimation of L3 muscle measurement in manual delineation compared with automatic threshold selection. This result, similar to that of Swartz et al., can possibly be explained by the L3 muscles’ anatomical complexity; L3 muscles form a sophisticated region of interest that must be delineated, requiring third-party programs to measure [12]. By contrast, manual delineation and threshold selection resulted in consistent M-SMI results. We propose that because the masticatory muscle is well demarcated with less myosteatosis, this leads to similar results for both measurement methods. On the basis of these findings, we suggest that M-SMI measurement is less influenced by different measurement methods and should be given priority consideration for use in patients with HNC.

This study has the following limitations. The retrospective nature of our study means that our single-institute study design and relatively small sample size might have resulted in a degree of selection bias. Typically, patients with advanced HNC undergo PET-CT scans; therefore, we investigated whether patient selection contributed to biased results by enrolling patients with trauma as controls. We observed no difference between groups in the correlation between the M-SMI and L3-SMI, and the results of our study may be extrapolated to both patients with HNC and healthy individuals.

## Conclusions

The current study demonstrated the robust association between the M-SMI assessed at the mandibular notch level and L3 muscle measurements, suggesting that M-SMI measurements obtained from head and neck CT scans could be a readily available and feasible marker of whole-body SMM in patients with HNC. M-SMI measurements are less likely to be affected by interference from malignant lymphadenopathy or different measurement methods, and a low M-SMI (<5.5) independently predicted sarcopenia. For clinicians measuring whole-body SMM and screening for sarcopenia in patients with HNC, the masticatory assessment is cost-effective and does not entail additional radiation exposure. Further prospective and large-scale studies are warranted to validate the present results and ascertain the value of M-SMI in prognostication for patients with HNC.

## Supporting information

S1 Appendix The data of muscle measurements. (XLSX)
