# Correction: Masticatory muscle index for indicating skeletal muscle mass in patients with head and neck cancer

**DOI:** 10.1371/journal.pone.0304880

**Published:** 2024-05-31

**Authors:** Sheng-Wei Chang, Yuan-Hsiung Tsai, Cheng-Ming Hsu, Ethan I. Huang, Geng-He Chang, Ming-Shao Tsai, Yao-Te Tsai

In the Ethical considerations subsection of Materials and Methods, the Institutional Review Board number is incorrect. The correct number is: 201900335B0.
